# The famous cultivated mushroom Bailinggu is a separate species of the *Pleurotus eryngii* species complex

**DOI:** 10.1038/srep33066

**Published:** 2016-09-15

**Authors:** Mengran Zhao, Jinxia Zhang, Qiang Chen, Xiangli Wu, Wei Gao, Wangqiu Deng, Chenyang Huang

**Affiliations:** 1 Institute of Agricultural Resources and Regional Planning, Chinese Academy of Agricultural Sciences, Beijing, China; 2 Key Laboratory of Microbial Resources, Ministry of Agriculture, Beijing, China; 3 Guangdong Institute of Microbiology, Guangzhou, China

## Abstract

The mushroom of the genus *Pleurotus* in western China, called Bailinggu, is a precious edible fungus with high economic value. However, its taxonomical position is unclear. Some researchers regard it as a variety of *P. eryngii*, namely *P. eryngii* var. *tuoliensis*, whereas others consider it to be a subspecies of *P. eryngii*, viz. *P. eryngii* subsp. *tuoliensis*. A total of 51 samples representing seven genetic groups of the genus *Pleurotus* were subjected to a phylogenetic analysis of partial sequences of the translation elongation factor 1 alpha gene (*ef1a*), the RNA polymerase II largest subunit gene (*rpb1*), the RNA polymerase II second largest subunit gene (*rpb2*) and nuc rDNA internal transcribed spacers (ITS). Our data indicate that the mushroom Bailinggu is a lineage independent of *P. eryngii* and should be lifted as its own species, namely *P. tuoliensis*. In addition, its known distribution range consists of both western China and Iran.

The mushroom from the genus *Pleurotus* (Fr.) P. Kumm., which is found in western China and commercially called Bailinggu, is a precious edible fungus with a white fruiting body and crisp texture. Wild Bailinggu is usually associated with plants of the genus *Ferula* L. of the family Umbelliferae^1^. Therefore, its geographical distribution is closely related to that of the plants of the genus *Ferula* and is restricted to Yumin, Tuoli, Qinghe, Mulei and Shihezi of the Xinjiang Autonomous Region in western China at an altitude of 790–1400 m^2^. The first collection of Bailinggu was made by Mou on the roots of *Ferula krylovii* Korov. in Tuoli^2^, and then it was collected on *F. ferulaeoides* (Steud) Korov. in Mulei^3^. Based on its morphological features, host and altitude, Bailinggu was described as a new variety of *Pleurotus eryngii* (DC. ex Fr.) Quél., namely, *P. eryngii* var. *tuoliensis* Mou^3^.

The early studies on the taxonomy of Bailinggu were mainly based on morphological characteristics, leaving many open questions and controversies. Three different Latin names were successively used to name the wild mushroom Bailinggu. Huang^4^ considered Bailinggu to be a variety of the *P. eryngii* species complex and named it *P. eryngii* var. *nebrodensis* (Inzenga) Sacc. Mao^5^ regarded Bailinggu as another independent *Pleurotus* species *P. nebrodensis* (Inzenga) Quél., which was originally described from the Italian island Sicily. Moreover, some other mycologists regarded Bailinggu as *P. eryngii* var. *ferulae* (Lanzi) Sacc.

With the development of molecular techniques, mycologists began to investigate the taxonomic status of Bailinggu using molecular methods. The results obtained from ITS sequence and IGS-RFLP analyses indicated that Bailinggu from China was a different species from *P. eryngii* var. *ferulae*, which resulted in it being erroneously regarded as *P. nebrodensis* by Zhang *et al.*^6^. However, *P. nebrodensis*, which weakly parasitize *Prangos ferulacea* (L.) Lindl., is uniquely associated with *Prangos* Lindl. plants^7^. In contrast, Bailinggu from western China is associated with plants of the genus *Ferula* there. Furthermore, Kawai *et al.*^8^ found that Chinese Bailinggu was distinct from Sicilian *P. nebrodensis* based on ITS and IGS1 analyses, and the results indicated that Chinese Bailinggu evolved independently in China. The study conducted by Kawai *et al.*^8^ suggested the scientific name of *P. eryngii* var*. tuoliensis*. The phylogeny of the *P. eryngii* species complex based on the results of ITS and *ef1α* analyses supports the viewpoint of Kawai and his colleagues^9^,^10^. Based on mating experiments and ITS and IGS1 sequence analyses, Zervakis *et al.*^11^ upgraded its taxonomic status to subspecies, and treated Bailinggu as *P. eryngii* subsp. *tuoliensis* (C.J. Mou) Zervakis & Venturella.

Many studies have shown that single-copy protein encoding regions are more suited for revealing the relationships of closely related species^12^. Based on an analysis of *ef1a* and *rpb2* sequence data, Rodriguez Estrada *et al.*^13^ treated *P. eryngii* var. *nebrodensis* as an independent species, which is consistent with the viewpoint of Venturella^7^. The present study include a phylogenetic analysis of several genetic groups in the *Pleurotus* genus that was implemented using four nuclear DNA fragments (*ef1a*, *rpb1*, *rpb2* and ITS) to infer the taxonomic status of Bailinggu from western China and its relationships with other related species. The phylogenetic species were then delimited in this study according to the genealogical concordance phylogenetic species recognition (GCPSR) criterion^14^.

## Results

### Morphology

**Figure 1:**
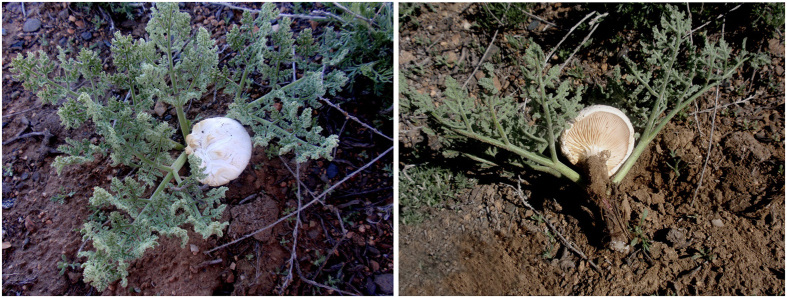
Basidiomata of *Pleurotus tuoliensis* (GDGM 27082)

**Figure 2:**
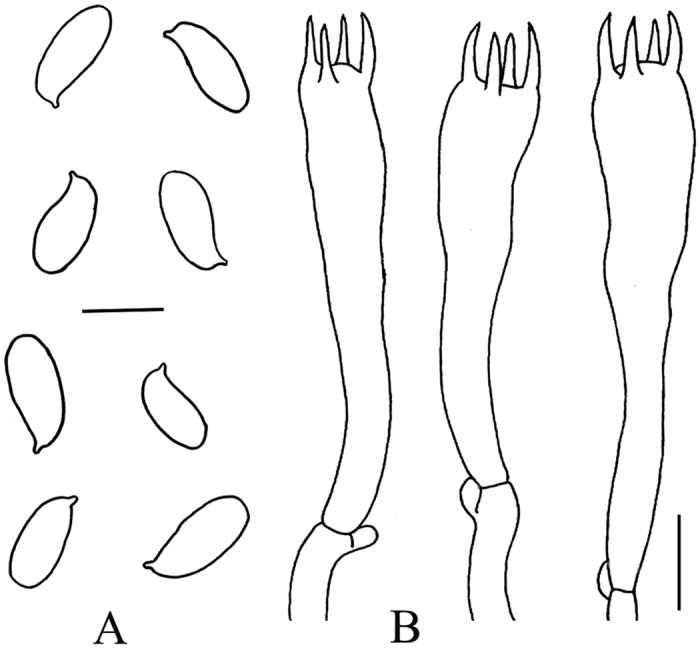
Microscopic characters of *Pleurotus tuoliensis* (GDGM 27082). (**A**) Basidiospores; (**B**) Basidia. Bars: A and B = 10 μm.

Pileus cochleariform to flabelliform, margin inrolled, convex; surface white, with cream-colored spots, with cracks and indistinct scales; flesh white, thick. Gills white, crowded, decurrent, 1–2 mm in width. Stipe lateral, solid, white, attenuate downwards (Fig. 1). Spore (9) 10–14 × (4.2) 5–6 μm, Q = 2.0–2.5 (Q = 2.2 ± 0.21), oblong-elliptic to elliptic, colorless and hyaline (Fig. 2A). Basidia 30–45 (50) × 7–9 μm, clavate, hyaline, thin-walled, four-spored (Fig. 2B).

### Phylogenetic analysis and phylogenetic species recognition

Both *ef1a* (except CCMSSC 04235) and *rpb2* (except CCMSSC 00929) were successfully amplified from 50 samples. A gene fragment of *rpb1* was obtained from only 47 samples. After sequence alignment, editing and trimming, 525-bp, 1152-bp and 1093-bp segments, which contained 95, 307 and 102 parsimony informative sites, respectively, remained for phylogenetic analysis. The ITS dataset consisted of 50 sequences (with the exception of CCMSSC 00761) generated in this study and 48 related ITS sequences retrieved from GenBank (Table S1). The sequence alignment comprised 577 nucleotide positions in the ITS region used for the phylogenetic analysis.

**Figure 3:**
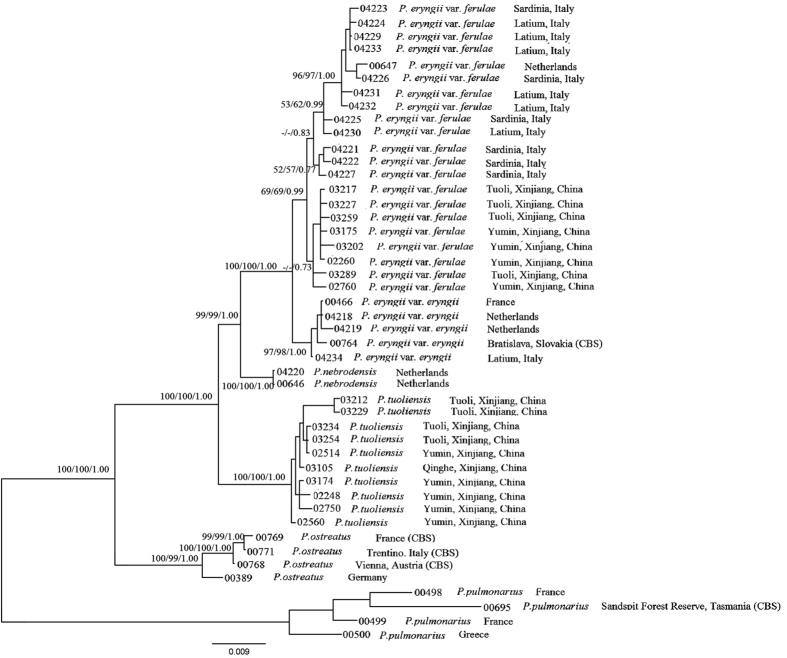
Phylogenetic tree of *Pleurotus* species inferred from maximum likelihood (ML) analysis based on the combined dataset (*ef1a*, *rpb2*, and *rpb1*). Only maximum parsimony bootstraps (PB) and maximum likelihood bootstraps (LB) over 50% and Bayesian posterior probabilities (PP) over 0.70 are reported on the branches.

The phylogenetic trees that were reconstructed with three independent gene fragments (*ef1a*, *rpb2* and *rpb1*) and inferred from a maximum likelihood (ML) analysis together with maximum likelihood bootstraps (LB), maximum parsimony bootstraps (PB) and Bayesian posterior probabilities (PP) are shown in Figs S1–S3, respectively. The three phylogenetic trees share the same topology. The phylogenetic tree obtained from the ML analysis with LB, PB and PP support based on the combined dataset (*ef1a*, *rpb2* and *rpb1*) is shown in Fig. 3. Six major clades supported with high bootstrap values and posterior probabilities could be inferred, corresponding with samples of var. *ferulae*, var. *eryngii*, *P. nebrodensis*, *P. tuoliensis* (Bailinggu), *P. ostreatus* (Jacq.) P. Kumm. and *P. pulmonarius* (Fr.) Quél. Our results identified the mushroom Bailinggu as a monophyletic group supported by a bootstrap value of 100% and a posterior probability value of 1.00. According to the GCPSR criterion, the mushroom Bailinggu should be recognized as an independent phylogenetic species based on the fact that it is highly divergent from its sibling groups.

### Phylogenetic relationships among Bailinggu and its related species

**Figure 4:**
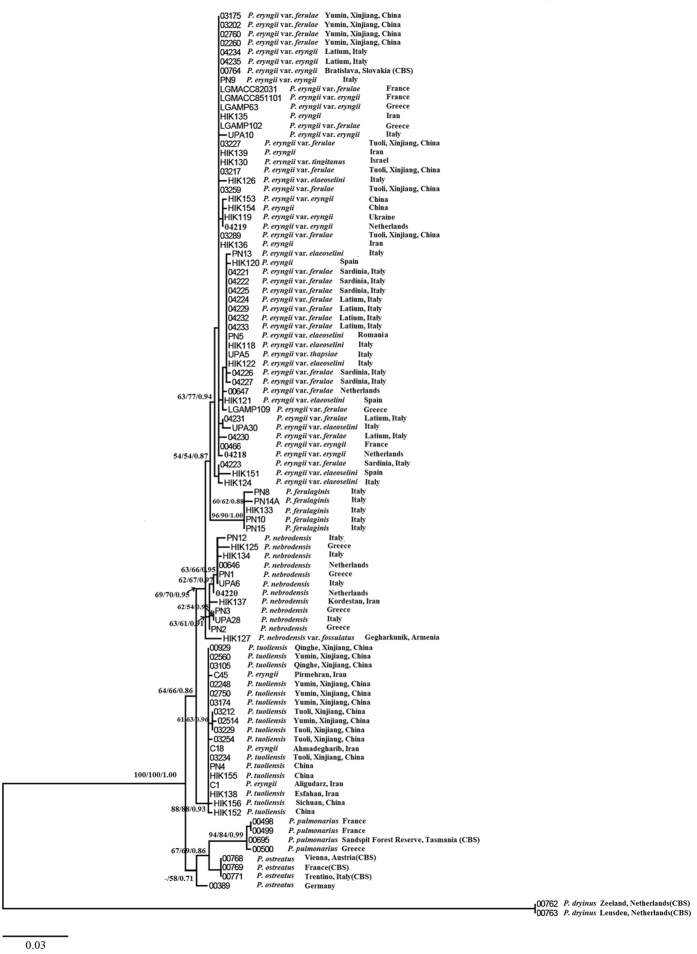
Phylogenetic tree of *Pleurotus* species inferred from maximum likelihood (ML) analysis based on ITS sequences. Only maximum parsimony bootstraps (PB) and maximum likelihood bootstraps (LB) over 50% and Bayesian posterior probabilities (PP) over 0.70 are reported on the branches.

ML, maximum parsimony (MP) and Bayesian algorithm (BA) analyses based on the ITS dataset yielded similar tree topologies with some differences in bootstrap and posterior probability values. The tree inferred from the ML analysis is shown in Fig. 4. A phylogenetic reconstruction based on the ITS dataset clustered the *P. eryngii* species complex samples into four major clades, which are supported with moderate bootstrap and high posterior probability values. One clade consists of the varieties *eryngii*, *ferulae*, *elaeoselini* Venturella, Zervakis & La Rocca, *thapsiae* Venturella, Zervakis & Saitta, and *tingitanus* Lewinsohn. The other three clades correspond to *P. ferulaginis* Zervakis, Venturella & Cattarossi from Italy, *P. nebrodensis* from Europe and Asia, and *P. tuoliensis* (Bailinggu) from Asia. The samples of Bailinggu form a monophyletic group in the ITS tree, which exhibits the furthest genetic distance from the other groups of the *P. eryngii* species complex. These results are consistent with those obtained based on each single-copy protein-encoding gene. The phylogenetic relationships among *P. eryngii* var. *eryngii*, *P. eryngii* var. *ferulae*, *P. eryngii* var. *elaeoselini*, *P. eryngii* var. *thapsiae*, and *P. eryngii* var. *tingitanus* obtained using the ITS dataset remain resolved.

## Discussion

Bailinggu is one of the most widely cultivated mushrooms in China. Recently, this species has been involved in the researches of genetic diversity evaluation^15^,^16^, temperature response mechanism^17^,^18^,^19^,^20^, fructification mechanism^21^, and bioactive substance exploitation^22^. However, the most essential information on the taxonomic status of Bailinggu and its phylogenetic relationships with its sibling species remain uncertain.

The mushrooms from *Pleurotus* genus that grow on the roots and stems of Umbelliferae plants belong to the *P. eryngii* species complex. The morphological characteristics of Bailinggu from western China conform to those of the *P. eryngii* species complex^23^. The morphological differences between Bailinggu and its related species are shown in Table 1. The pileus color of *P. eryngii* var. *eryngii* ranges from brown and beige-brown to light beige, whereas the pileus color of *P. eryngii* var. *ferulae* from Europe ranges from grey-brown to slate grey to beige brown. The macro-morphological characteristics of Bailinggu are similar to those of *P. nebrodensis*, but the basidiospores of Bailinggu are slightly smaller than those of *P. nebrodensis.* The pileus color of *P. eryngii* var. *ferulae* from China is brown to white, therefore, it is not possible to distinguish Bailinggu from *P. eryngii* var. *ferulae* from China based exclusively on their macroscopic and microscopic characteristics.

The intersterility criterion is a derivative of the biological species criteria. Many cryptic species, such as the *Armillaria mellea* (Vahl) P. Kumm.^24^, have recently been recognized using this criterion. Previous mating compatibility tests of the *P. eryngii* species complex did not indicate any complete reproductive isolation among the genetic groups within the *P. eryngii* species complex. The mating rate between *P. eryngii* var. *eryngii* and *P. eryngii* var. *ferulae* was the highest, with a value of 98%^8^ or 93%^10^, but those between *P. nebrodensis* and *P. eryngii* var. *eryngii* and between *P. nebrodensis* and were significantly lower, with values of 6–18%^25^. Very few mating tests have been performed between Bailinggu and other genetic groups. According to the previous studies, Chinese Bailinggu showed much higher compatibility with *P. eryngii* var. *eryngii* (65%) and *P. eryngii* var. *ferulae* (82%) than with *P. nebrodensis* (15%) and *P. ferulaginis* (11%)^8^,^11^. This indicates that Bailinggu might be closer to *P. eryngii* var. *eryngii* and *P. eryngii* var. *ferulae*. However, some evidence that many fungi genetically isolated in nature retain the ancestral character of interbreeding^14^. Hilber^26^ found that *P. eryngii* var. *eryngii* and *P. eryngii* var. *ferulae* could mate with each other in the laboratory, but they appear to be reproductively isolated in the field and are associated with specific host plants.

The results of this study based on molecular data showed that Bailinggu is a separate phylogenetic species instead of a variety or subspecies of the *P. eryngii* complex according to the GCPSR criterion, although this mushroom retains high intercompatibility with *P. eryngii* var. *eryngii* and *P. eryngii* var. *ferulae* in the laboratory. A similar observation was found in a study of the *P. ostreatus* complex. Three intersterility groups or biological species (I, II, and VI) in the *P. ostreatus* complex were found to contain more than one phylogenetic species^27^. A phylogenetic reconstruction based on the ITS dataset and the combined dataset revealed that the genetic distance between Bailinggu and *P. eryngii* (var. *eryngii*, var. *ferulae*, var. *elaeoselini*, var. *thapsiae*, var. *tingitanus*) was greater than those between Bailinggu and *P. ferulaginis* and between Bailinggu and *P. nebrodensis*. This result is in conflict with the previous findings in the mating tests. Considering the geographical isolation of Bailinggu in nature, the results inferred from molecular data are more acceptable because DNA sequence divergence, be it allopatric or sympatric, might occur much earlier than the evolution of intersterility^28^,^29^.

Previous research using sequence analyses of ITS and IGS1 showed that Bailinggu is a phylogenetic sister group to *P. eryngii*^11^. However, our study indicates that *P. ferulaginis* is much more similar to *P. eryngii* in terms of not only morphology, distribution, and ecology but also DNA divergence. The phylogenetic analysis revealed that Bailinggu is a sister group to the *eryngii-ferulaginis-nebrodensis* clade and is not closely related to the other genetic groups of the *P. eryngii* species complex.

Reproductive isolation caused by host specialization is often observed in basidiomycetes, particularly plant pathogenic fungal species^30^. To the best of our knowledge, the *P. eryngii* species complex has developed a certain degree of host specificity. To detect whether the relationships among the genetic groups of the species complex correlate with those among their hosts, the phylogeny of the relevant hosts was reconstructed based on ITS1 and ITS2 sequences retrieved from GenBank (Fig. S4). The results showed that the *eryngii*, *ferulae*, *elaeoselini*, *thapsiae*, and *tingitanus* varieties are so closely related genetically that they could not be distinguished by ITS analysis, but the relationship among hosts of *P. eryngii* var. *eryngii, P. ferulaginis*, and *P. nebrodensis* is markedly closer. In contrast, the genetic relationships of Bailinggu with the *ferulae*, *elaeoselini*, *thapsiae*, and *tingitanus* varieties are distant, but the genetic relationships among their hosts are close, indicating that hosts might not be the main reason for the divergence of Bailinggu from other genetic groups. Its long geographical isolation might be the main reason for the distant genetic relationship among Bailinggu and other genetic groups.

*Pleurotus eryngii*, *P. ferulaginis* and *P. nebrodensis* are mainly distributed in the Mediterranean and surrounding areas, whereas recent studies found that *P. eryngii* and *P. nebrodensis* also occur in Asia^11^. The distributions of the two mushrooms are wide and continuous, but there is very limited information on the distribution of Bailinggu. The samples of Bailinggu used in the present study were mostly from western China, and partly from Iran^11^,^31^. The main distribution area of Bailinggu in China is located far from the distribution areas of other genetic groups with the exception of *P. eryngii* var. *ferulae* from China. There are no obvious differences in morphological characteristics or habitat between Bailinggu and *P. eryngii* var. *ferulae* from China. However, a sequence analysis showed a remarkable difference between them in terms of DNA sequence, which is consistent with previous results^6^. What efficient prezygotic barriers that maintain the separation of both gene pools will require further study. The pileus color of *P. eryngii* var*. ferulae* from China is different from that of *P. eryngii* var*. ferulae* from Europe. Moreover, the phylogenetic analysis showed that they cluster according to their geographical origins even though they still belong to the same genetic group. Geographical isolation and differences in biotope would likely lead to increasing divergence of an individual population to enhance differentiation^32^,^33^.

## Conclusion

This study, which involved multiple or independent DNA gene fragment analyses in combination with a morphological analysis, showed that Bailinggu is highly divergent from its related groups at the DNA level but presents no significant differences in morphology or mating incompatibility. According to the GCPSR criterion, Bailinggu is an independent phylogenetic species in the *P. eryngii* complex, and based on its geographical isolation in nature, *P. eryngii* var. *tuoliensis* or *P. eryngii* subsp. *tuoliensis* should be upgraded to an independent species, and *P. tuoliensis* should be the scientific name for this mushroom. The taxonomic treatment is as follows:

***Pleurotus tuoliensis*** (C.J. Mou) M.R. Zhao & J.X. Zhang, comb. nov. & stat. nov.

Fungal Name No.: FN570249.

Basionym: *Pleurotus eryngii* var. *tuoliensis* C.J. Mou, Acta Mycol. Sin. 6(3): 153 (1987) [MycoBank No.:133079]; *Pleurotus eryngii* subsp. *tuoliensis* (C.J. Mou) Zervakis & Venturella, Fungal Biology 118: 826 (2014) [MycoBank No.:807241].

Specimen examined: GDGM 27082 (Table 2).

## Materials and Methods

### Taxon sampling

Fifty-one specimens representing seven different genetic groups of *Pleurotus* were used in this study (Table 2). These samples were obtained through field collection, donation, culture exchange and purchasing. Pure cultures of all samples were deposited at the China Center for Mushroom Spawn Standards and Control (CCMSSC).

### Morphological observation

The morphological characteristics of fresh fruitbodies were observed and recorded in the field. The samples were dried at 40–50 °C, and microscopic features were observed with a light microscope. The size of the basidiospores was described in the form of (a)b–c(d), and 90% of the measurements were within the range of b and c; a and d (in the parentheses) are the minimum and maximum of the measurements, respectively, whereas the quotient (Q) of their dimensions was calculated as the ratio of the spore length (arithmetic average of all spores) to the spore width (arithmetic average of all spores).

### DNA extraction, amplification and sequencing

Total DNA was extracted using a DP305-Plant Genome Extraction Kit (Tiangen, China). PCR amplifications were conducted using the following primer pairs: EF595F/EF116OR for the portion of the *ef1a* gene, fRPB2 5F/bRPB2 7.1R, b6.9F/b11R1 for the fragment of the *rpb*2 gene^12^, RPB1 2F (5′ ATTGCGGGCGACTAAAGG 3′) and RPB1 5R (5′ CTGCTCAAACTCGGAGATAA 3′) for the part of the *rpb*1 gene, and ITS1/ITS4^34^ for the ITS region. Each amplification reaction system contained approximately 20 ng of DNA template, 0.2 mM dNTPs, 0.5 mM each primer, and 1 U of Ex *Taq* DNA polymerase (TaKaRa, Japan) in a final volume of 20 μL. The PCR was conducted using the following program: 94 °C for 4 min followed by 35 cycles of 94 °C for 50 s, 55 °C for 50 s, and 72 °C for 1 min. The reaction was completed by incubation at 72 °C for 10 min. The amplified products were separated by electrophoresis on 1.2% agarose gels and stained with ethidium bromide. Sequencing was performed by BGI Co., Ltd (Beijing, China). The PCR products from each sample that failed to yield direct sequencing results were cloned using a pGEM-T easy cloning kit (Promega, USA) and transformed into DH5α component *Escherichia coli* cells. Ten random transformed *E. coli* colonies were selected for sequencing, and the sequence data were deposited in GenBank (Table 2).

### Sequence alignments

Each DNA sequence was assembled and edited manually if needed. Sequence alignments were performed using the MUSCLE algorithm in MEGA 5.0^35^. Different alignments were performed for different analytical purposes. Multiple or independent DNA gene fragments (*ef1a*, *rpb*2 and *rpb1*) were used to reconstruct the phylogeny of mushrooms of the genus *Pleurotus* to infer the taxonomic status of the Chinese Bailinggu. For the *Pleurotus* genus, more ITS sequences than sequences of the other three genes were readily available in GenBank, and the relationships among Bailinggu and its sibling species were further investigated using the ITS dataset.

### Phylogenetic analysis

Phylogenetic reconstructions using *ef1a*, *rpb1*, *rpb2*, the combined data set of the three genes, and ITS were performed using MP, ML and BA. The MP analyses were performed with PAUP* 4.0b10^36^. Heuristic searching with TBR branch swapping was implemented with 1000 random-addition sequence replicates. The bootstrap analysis was conducted with 1,000 replicates using the heuristic search^37^. ML analyses were conducted in PHYML3.0^38^, and the bootstrap analysis was performed with 1000 replicates. BA analyses were run using MrBayes3.1.2^39^. The Markov Chain Monte Carlo (MCMC) algorithm^40^ was utilized to calculate the Bayesian posterior probabilities. Four Markov chains were run for 5,000,000 generations with the trees sampled every 1000^th^ generation. The average standard deviation of split frequencies was restricted to less than 0.01. The first 25% trees were discarded as burn-in. The optimum substitution model for each dataset was estimated by jModelTest^41^ according to the Corrected Akaike Information Criterion (AIC)^42^ for the ML analyses and the Bayesian information criterion (BIC)^43^ for the Bayesian analyses. For the ML analyses, the optimal substitution models for the four partitions determined using the AIC were as follows: TIM1 + G for *ef1a* and *rpb1*, SYM + G for *rpb2*, and TPM2uf + G for ITS. The Bayesian analyses were performed with the following selected substitution models: TrNef + G for *ef1a* and *rpb2*, TPM1 + G for *rpb1*, and TPM2uf  + G for ITS. The samples without available sequences were not used in the phylogenetic reconstructions.

### Phylogenetic species determination

The phylogenetic species were delimited in this study according to the genealogical concordance phylogenetic species recognition (GCPSR) criterion. Using this method, phylogenetic species were recognized as genealogically exclusive under GCPSR if they were concordantly supported by multiple independent loci^14^.

## Additional Information

**How to cite this article**: Zhao, M. *et al.* The famous cultivated mushroom Bailinggu is a separate species of the *Pleurotus eryngii* species complex. *Sci. Rep.* **6**, 33066; doi: 10.1038/srep33066 (2016).

## Supplementary Material

Supplementary Information

## Figures and Tables

**Table 1:** The distinctive discriminating morphological characters for the *Pleurotus eryngii* species complex.

Source	Species or variety	Spore size (μm)		Pileus color	Gill	Stipe position
		Length	Width			
Zervakis *et al.*^25^	*P. eryngii* var. *eryngii*	9.1–13.5	4.8–6.7	Brown-red brown, warm brown, light beige to beige brown	Decurrent, cream to light beige, anastomoses	Central to eccentric
	*P. eryngii* var. *ferulae*	9.6–13.8	4.7–6.9	Grey-brown to slate grey to beige brown	Decurrent, cream to light beige, anastomoses	Central to eccentric
	*P. nebrodensis*	13.2–17.4	5.5–8.2	Light ivory to cream	Deeply decurrent, whitish to pale yellow reticulum at stipe	Central to eccentric, radiating
Kawai *et al.*^8^	*P. eryngii* var. *ferulae*	10–12	4.5–5.6	Brown, pale brown	Decurrent, pale brown, pale yellow brown	Central, white
	*P. nebrodensis*	11–16	5–8	White, centrally tinged pale brown	Decurrent, cream	Central, white
	Bailinggu	9–14	4–6	White	Decurrent, dull white, cream	Central, eccentric, white
Zervakis *et al.*^11^	Bailinggu	8.7–14.3	4.5–6.3	White to cream	×	×
	*P. ferulaginis*	11.0–16.0	4.0–5.5	Whitish to ochraceous to beige to brown	White to cream to ivory	Decurrent
Teng^44^	*P. eryngii* var. *ferulae*	12–14	5–6	Brown, to white gradually	Decurrent, white, light yellow	Eccentric, white
Ying *et al.*^45^	*P. eryngii* var. *ferulae*	12–14	5–6	Brown, to white gradually	Decurrent, white to light yellow	Eccentric, white
Mao^5^	Bailinggu	9–13.5	4.5–5.5	White	Decurrent, white	Lateral, eccentric, white

The crosses indicate that the trait has not been described.

**Table 2:** The information and GenBank accession numbers of the *Pleurotus* samples used in this study.

Strain No. (CCMSSC)	Taxa	Geographic origin	ITS	*ef1α*	*rpb2*	*rpb1*
03105	*P. tuoliensis*	Qinghe, Xinjiang, China	KU612906	KU612970	KU612991	KU612948
00929	*P. tuoliensis*	Qinghe, Xinjiang, China	KU612907	KU612971	×	×
03212	*P. tuoliensis*	Tuoli, Xinjiang, China	KU612908	KU612972	KU612992	KU612949
03229	*P. tuoliensis*	Tuoli, Xinjiang, China	KU612909	KU612973	KU612993	KU612950
03234	*P. tuoliensis*	Tuoli, Xinjiang, China	KU612910	KU612974	KU612994	KU612951
03254	*P. tuoliensis*	Tuoli, Xinjiang, China	KU612911	KU612975	KU612995	KU612952
03174	*P. tuoliensis*	Yumin, Xinjiang, China	KU612912	KU612976	KU612996	KU612953
02248	*P. tuoliensis*	Yumin, Xinjiang, China	KU612913	KU612977	KU612997	KU612954
02514*	*P. tuoliensis*	Yumin, Xinjiang, China	HM777041	KU983512	KU983514	KU983513
02560	*P. tuoliensis*	Yumin, Xinjiang, China	KU612914	KU612978	KU612998	KU612955
02750	*P. tuoliensis*	Yumin, Xinjiang, China	KU612915	KU612979	KU612999	KU612956
03217	*P. eryngii* var. *ferulae*	Tuoli, Xinjiang, China	KU612916	KM000984	KU613000	KR493299
03227	*P. eryngii* var. *ferulae*	Tuoli, Xinjiang, China	KU612917	KM000995	KU613001	KR493310
03259	*P. eryngii* var. *ferulae*	Tuoli, Xinjiang, China	KU612918	KM000989	KU613002	KR493304
03289	*P. eryngii* var. *ferulae*	Tuoli, Xinjiang, China	KU612919	KM001005	KU613003	KR493320
03175	*P. eryngii* var. *ferulae*	Yumin, Xinjiang, China	KU612920	KM000979	KU613004	KR493294
03202	*P. eryngii* var. *ferulae*	Yumin, Xinjiang, China	KU612921	KM000954	KU613005	KR493269
02760	*P. eryngii* var. *ferulae*	Yumin, Xinjiang, China	KU612922	KM000962	KU613006	KR493277
02260	*P. eryngii* var. *ferulae*	Yumin, Xinjiang, China	KU612923	KM000935	KU613007	KR493250
00647	*P. eryngii* var. *ferulae*	Netherlands	KU612924	KU612980	KU613008	KU612957
04221	*P. eryngii* var. *ferulae*	Sardinia, Italy	KU612925	KR493212	KU613009	KR493322
04222	*P. eryngii* var. *ferulae*	Sardinia, Italy	KU612926	KR493213	KU613010	KR493323
04223	*P. eryngii* var. *ferulae*	Sardinia, Italy	KU612927	KR493214	KU613011	KR493324
04225	*P. eryngii* var. *ferulae*	Sardinia, Italy	KU612928	KR493215	KU613012	KR493325
04226	*P. eryngii* var. *ferulae*	Sardinia, Italy	KU612929	KR493216	KU613013	KR493326
04227	*P. eryngii* var. *ferulae*	Sardinia, Italy	KU612930	KR493217	KU613014	KR493327
04224	*P. eryngii* var. *ferulae*	Latium, Italy	KU612931	KR493218	KU613015	KR493328
04229	*P. eryngii* var. *ferulae*	Latium, Italy	KU612932	KR493219	KU613016	KR493329
04230	*P. eryngii* var. *ferulae*	Latium, Italy	KU612933	KU612981	KU613017	KU612958
04231	*P. eryngii* var. *ferulae*	Latium, Italy	KU612934	KR493220	KU613018	KR493330
04232	*P. eryngii* var. *ferulae*	Latium, Italy	KU612935	KR493221	KU613019	KR493331
04233	*P. eryngii* var. *ferulae*	Latium, Italy	KU612936	KR493222	KU613020	KR493332
04234	*P. eryngii* var. *eryngii*	Latium, Italy	KU612937	KU612982	KU613021	KU612959
04235	*P. eryngii* var. *eryngii*	Latium, Italy	KU612938	×	KU613022	KU612960
00466	*P. eryngii* var. *eryngii*	France	KU612939	KU612983	KU613023	KU612961
00764	*P. eryngii* var. *eryngii*	Bratislava, Slovakia (CBS 100.82)	EU424295	KR493223	KU613024	KR493336
04219	*P. eryngii* var. *eryngii*	Netherlands	KU612940	KR493224	KU613025	KR493337
04218	*P. eryngii* var. *eryngii*	Netherlands	KU612941	KU612984	KU613026	KU612962
04220	*P. nebrodensis*	Netherlands	KU612942	KU612985	KU613027	KU612963
00646	*P. nebrodensis*	Netherlands	KU612943	KU612986	KU613028	KU612964
00768	*P. ostreatus*	Vienna, Austria (CBS 102513)	KU612944	KR493225	KU613029	KR493333
00769	*P. ostreatus*	France (CBS 291.47)	EU424309	KR493226	KU613030	KR493334
00771	*P. ostreatus*	Trentino, Italy (CBS 375.51)	EU424310	KR493227	KU613031	KR493335
00389	*P. ostreatus*	Germany	KU612945	KU612987	KU613032	KU612965
00498	*P. pulmonarius*	France	KU612946	KU612902	KU613033	KU612966
00499	*P. pulmonarius*	France	EU424314	KU612903	KU613034	KU612967
00500	*P. pulmonarius*	Greece	KU612947	KU612904	KU613035	KU612968
00695	*P. pulmonarius*	Sandspit Forest Reserve, Tasmania, Australia (CBS 100130)	EU424311	KU612905	KU613036	KU612969
00761	*P. dryinus*	Harz, Germany (CBS 481.72)	×	KU612988	KU613037	×
00762	*P. dryinus*	Zeeland, Netherlands (CBS 724.83)	EU424293	KU612989	KU613038	×
00763	*P. dryinus*	Leusden, Netherlands (CBS 804.85)	EU424294	KU612990	KU613039	×

^*^The fruiting body of this strain was collected by Qiang Chen at Zhuanchang (altitude 735 m) of Yumin county in Xinjiang Autonomous Region on April 22, 2009. And now it was deposited at the herbarium of Guangdong institute of Microbiology, the herbarium No. was GDGM 27082.

The crosses indicate that sequences were not available. CBS (Centraalbureau voor Schimmelcultures, the Netherlands). CBS numbers are presented in parentheses.
