# Supplementary material for: The famous cultivated mushroom Bailinggu is a separate species of the Pleurotus eryngii species complex
Source: Sci Rep. 2016 Sep 15;6:33066. doi: 10.1038/srep33066 (PMC5024158; doi:10.1038/srep33066)
Supplement: Supplementary Information [file srep33066-s1.pdf]

The famous cultivated mushroom Bailinggu was a separate species of the *Pleurotus eryngii* species-complex

Mengran Zhao , Jinxia Zhang , Qiang Chen, Xiangli Wu, Wei Gao,

Wangqiu Deng, Chenyang Huang *

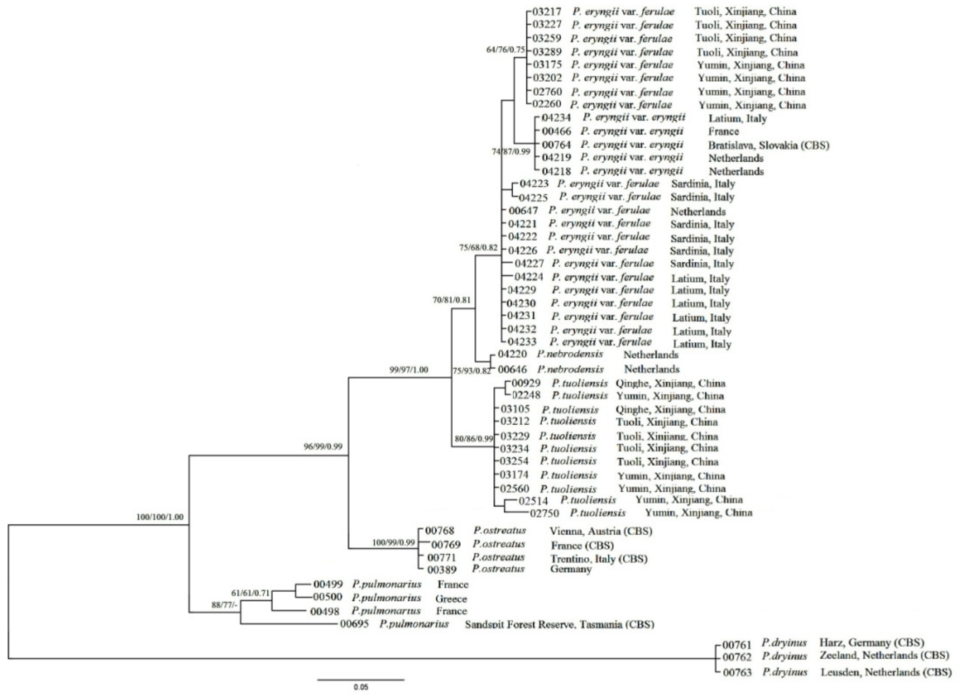

Figure S1 Phylogenetic tree of *Pleurotus* species inferred from maximum likelihood (ML) analysis based on *ef1α* sequences. Only maximum parsimony bootstraps (PB) and maximum likelihood bootstraps (LB) over 50%, and Bayesian posterior probabilities (PP) over 0.70 are reported on the branches.

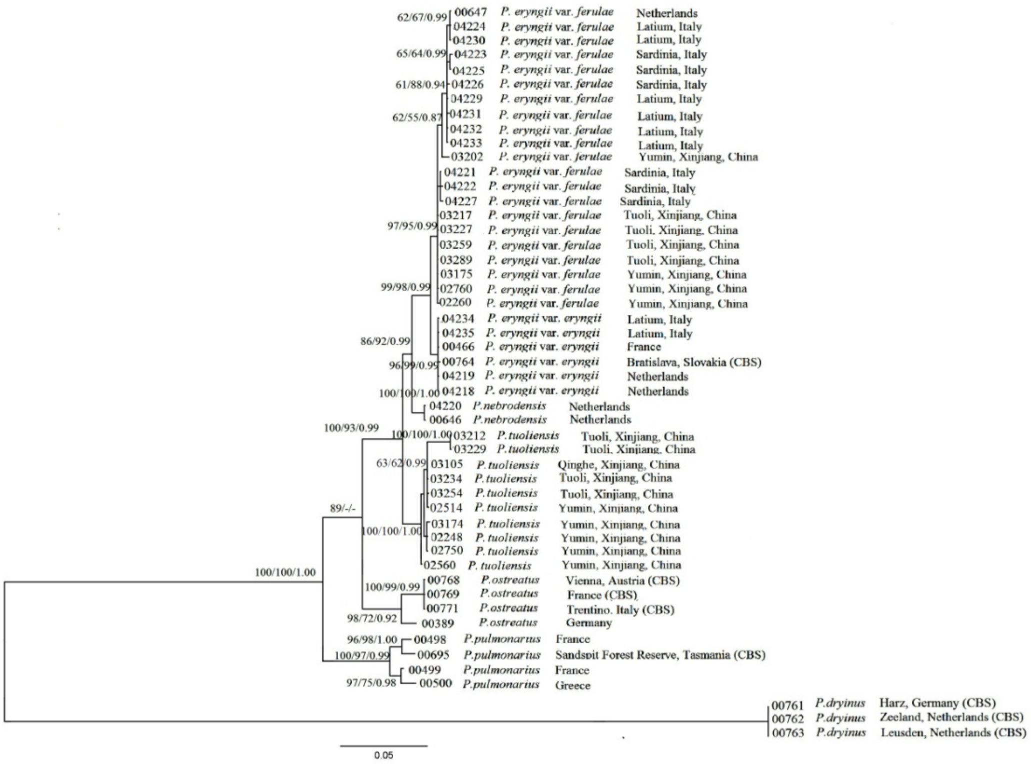

Figure S2 Phylogenetic tree of *Pleurotus* species inferred from maximum likelihood (ML) analysis based on *rpb2* sequences. Only maximum parsimony bootstraps (PB) and maximum likelihood bootstraps (LB) over 50%, and Bayesian posterior probabilities (PP) over 0.70 are reported on the branches.

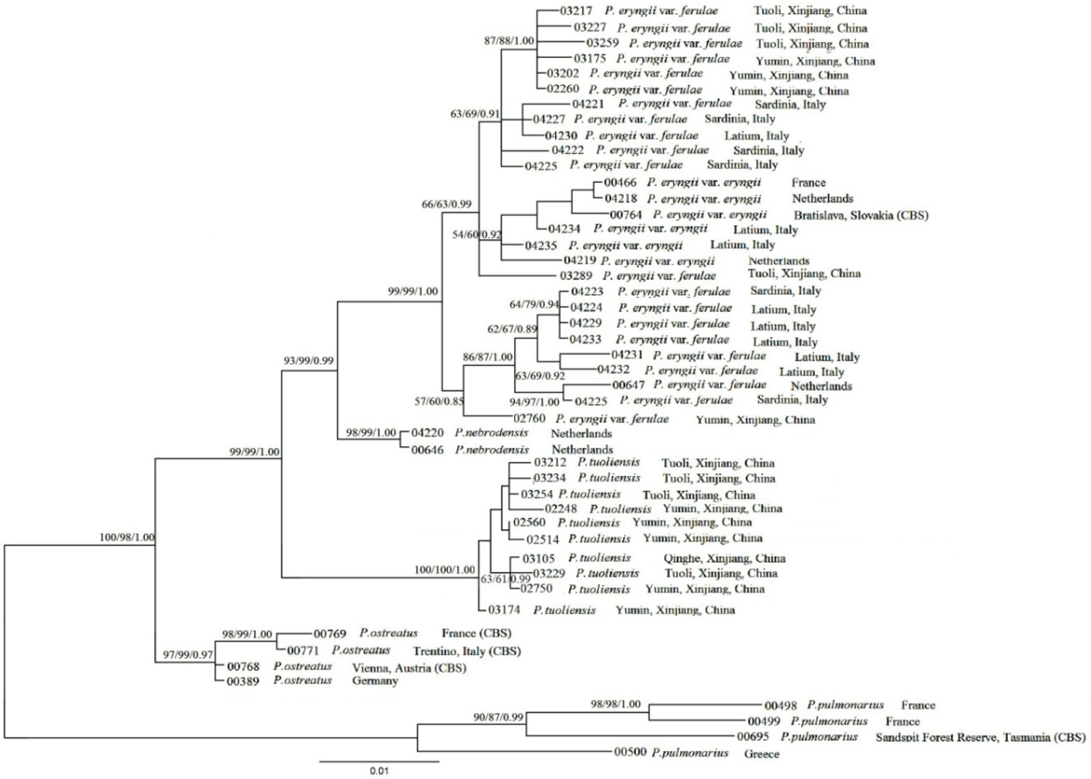

Figure S3 Phylogenetic tree of *Pleurotus* species inferred from maximum likelihood (ML) analysis based on *rpb1* sequences. Only maximum parsimony bootstraps (PB) and maximum likelihood bootstraps (LB) over 50%, and Bayesian posterior probabilities (PP) over 0.70 are reported on the branches.

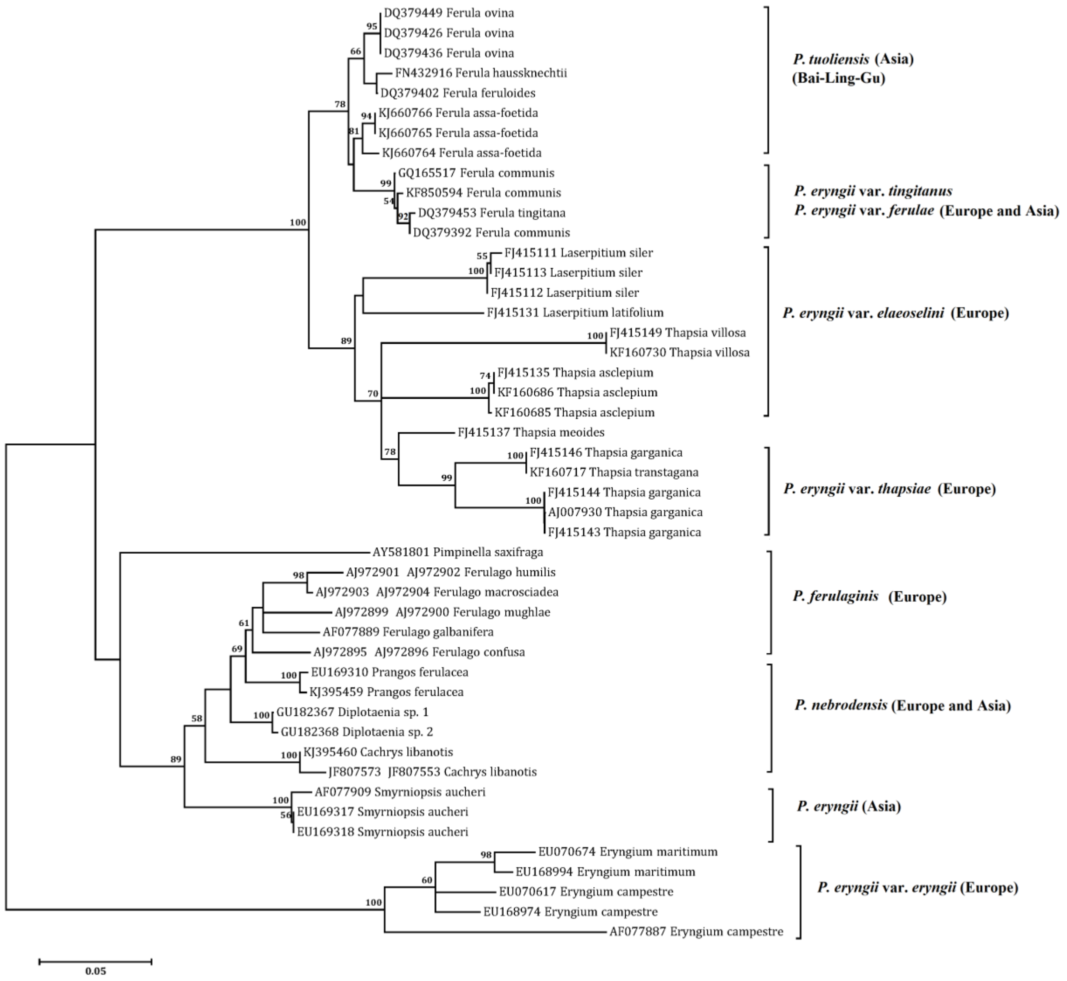

Figure S4 Molecular phylogenetic anaylsis of the hosts of *Pleurotus eryngii* species-complex by Maximum Likelihood method based on the sequences of ITS1 and ITS2 retrieved from GenBank. The analysis involved 47 nucleotide sequences. There were a total of 486 positions in the final dataset. The bootstrap consensus tree inferred from 1000 replicates is taken to represent the evolutionary history of the taxa analyzed. Branches corresponding to partitions reproduced in less than 50% bootstrap replicates are collapsed. The percentage of replicate trees in which the associated taxa clustered together in the bootstrap test is shown above the branches.

Table S1 The accession numbers of ITS sequences retrieved from GenBank in this study

Strain numbers	Taxa	Geographic origin	ITS Accession Number
HIK119	*P. eryngii* var. *eryngii*	Ukraine	HM998820
PN9	*P. eryngii* var. *eryngii*	Italy	KF743828
LGMACC851101	*P. eryngii* var. *eryngii*	France	HM998810
LGMAP63	*P. eryngii* var. *eryngii*	Greece	HM998811
HIK153	*P. eryngii* var. *eryngii*	China	HM998840
UPA10	*P. eryngii* var. *eryngii*	Italy	HM998817
HIK154	*P. eryngii*	China	HM998841
HIK139	*P. eryngii*	Iran	HM998837
HIK136	*P. eryngii*	Iran	HM998834
HIK135	*P. eryngii*	Iran	HM998833
HIK120	*P. eryngii*	Spain	HM998821
C45	*P. eryngii*	Pirmehran, Iran	FJ514590
C18	*P. eryngii*	Ahmadegharib, Iran	FJ514565
C1	*P. eryngii*	Aligudarz, Iran	FJ514549
HIK122	*P. eryngii* var. *elaeoselini*	Italy	HM998823
HIK126	*P. eryngii* var. *elaeoselini*	Italy	HM998827
PN13	*P. eryngii* var. *elaeoselini*	Italy	KF743831
HIK151	*P. eryngii* var. *elaeoselini*	Spain	HM998838
HIK124	*P. eryngii* var. *elaeoselini*	Italy	HM998825
PN5	*P. eryngii* var. *elaeoselini*	Romania	KF743824
HIK118	*P. eryngii* var. *elaeoselini*	Italy	KF743825
HIK121	*P. eryngii* var. *elaeoselini*	Spain	HM998822
UPA30	*P. eryngii* var. *elaeoselini*	Italy	HM998819
LGMACC820301	*P. eryngii* var. *ferulae*	France	HM998808
LGAMP102	*P. eryngii* var. *ferulae*	Greece	HM998813
LGAMP109	*P. eryngii* var. *ferulae*	Greece	HM998814
UPA5	*P. eryngii* var. *thapsiae*	Italy	HM998815
HIK130	*P. eryngii* var. *tingitanus*	Israel	HM998829
HIK133	*P. ferulaginis*	Italy	KF743826
PN8	*P. ferulaginis*	Italy	KF743827
PN10	*P. ferulaginis*	Italy	KF743829
PN14A	*P. ferulaginis*	Italy	KF743832
PN15	*P. ferulaginis*	Italy	KF743833
HIK134	*P. nebrodensis*	Italy	HM998832
PN3	*P. nebrodensis*	Greece	KF743822

UPA28	*P. nebrodensis*	Italy	HM998818
PN2	*P. nebrodensis*	Greece	KF743821
PN1	*P. nebrodensis*	Greece	KF743820
PN12	*P. nebrodensis*	Italy	KF743830
UPA6	*P. nebrodensis*	Italy	HM998816
HIK125	*P. nebrodensis*	Greece	HM998826
HIK137	*P. nebrodensis*	Kordestan, Iran	HM998835
HIK127	*P. nebrodensis* var *fossulatus*	Gegharkunik, Armenia	HM998828
HIK152	*P. tuoliensis*	China	HM998839
HIK155	*P. tuoliensis*	China	HM998842
HIK156	*P. tuoliensis*	Sichuan, China	HM998843
PN4	*P. tuoliensis*	China	KF743823
HIK138	*P. tuoliensis*	Esfahan, Iran	HM998836
